# Potential Second-Hits in Hereditary Hemorrhagic Telangiectasia

**DOI:** 10.3390/jcm9113571

**Published:** 2020-11-05

**Authors:** Carmelo Bernabeu, Pinar Bayrak-Toydemir, Jamie McDonald, Michelle Letarte

**Affiliations:** 1 Centro de Investigaciones Biológicas Margarita Salas, Consejo Superior de Investigaciones Científicas (CSIC) and Centro de Investigación Biomédica en Red de Enfermedades Raras (CIBERER), 28040 Madrid, Spain; 2 ARUP Institute for Clinical and Experimental Pathology, Salt Lake City, UT 84108, USA; pinar.bayrak-toydemir@aruplab.com; 3 Department of Pathology, University of Utah, Salt Lake City, UT 84132, USA; Jamie.mcdonald@hsc.utah.edu; 4 Department of Radiology, University of Utah, Salt Lake City, UT 84132, USA; 5 Molecular Medicine, Hospital for Sick Children, and Department of Immunology, University of Toronto, Toronto, ON M5G 0A4, Canada; michelle.letarte@sickkids.ca

**Keywords:** hereditary hemorrhagic telangiectasia (HHT), second-hit, arteriovenous malformation (AVM), endoglin, ALK1, Smad4, inflammation, shear stress, vascular injury, somatic mutation, cell adhesion, angiogenesis, vascular endothelial growth factor (VEGF), transforming growth factor beta (TGF-β)

## Abstract

Hereditary hemorrhagic telangiectasia (HHT) is an autosomal dominant genetic disorder that presents with telangiectases in skin and mucosae, and arteriovenous malformations (AVMs) in internal organs such as lungs, liver, and brain. Mutations in *ENG* (endoglin), *ACVRL1* (ALK1), and *MADH4* (Smad4) genes account for over 95% of HHT. Localized telangiectases and AVMs are present in different organs, with frequencies which differ among affected individuals. By itself, HHT gene heterozygosity does not account for the focal nature and varying presentation of the vascular lesions leading to the hypothesis of a “second-hit” that triggers the lesions. Accumulating research has identified a variety of triggers that may synergize with HHT gene heterozygosity to generate the vascular lesions. Among the postulated second-hits are: mechanical trauma, light, inflammation, vascular injury, angiogenic stimuli, shear stress, modifier genes, and somatic mutations in the wildtype HHT gene allele. The aim of this review is to summarize these triggers, as well as the functional mechanisms involved.

## 1. Clinical Characteristics of HHT

Hereditary hemorrhagic telangiectasia (HHT) is an autosomal dominant vascular disorder that exhibits age-related penetrance and extensive clinical variability, including intra-familial variability [1]. The characteristic vascular lesions range from 1 to 2 mm punctate mucocutaneous telangiectases to arteriovenous malformations (AVMs) several centimeters in diameter within visceral organs, particularly the lungs, liver, and brain. Telangiectases close to the surface of the skin and mucous membranes are fragile and frequently rupture and bleed upon slight trauma. Spontaneous and recurrent nose-bleeding (epistaxis) typically begins in mid-childhood and is the most common clinical manifestation; although occurring in over 90% of patients, the severity varies from an infrequent few drops to brisk bleeds multiple times daily. Gastrointestinal (GI) bleeding due to mucosal telangiectases affects approximately 25% of patients, almost always presenting after the age of 50. Many HHT patients have iron-deficiency anemia secondary to chronic bleeding of telangiectases, more often from nasal than GI lesions [2,3].

Solid organ AVMs are direct connections between artery and vein which bypass capillary beds and result in life-threatening complications more often related to the shunting of blood per se through these low resistance pathways, than to hemorrhage. For example, pulmonary AVMs (PAVMs), which occur in about 50% of HHT patients overall, result in high-flow continuous intrapulmonary right-to-left shunts with significant related risk for stroke or brain abscess [4,5]. The majority of PAVMs (70% or more) occur in HHT patients, but approximately 20% are acquired and associated with trauma, cardiothoracic surgery, hepatic cirrhosis, metastatic cancer, mitral stenosis, infection, amyloidosis, and chronic thromboembolic disease [6]. The frequency of hepatic vascular malformations was approximately 75% in two studies that systematically imaged the liver of affected individuals using computed tomography (CT) [7,8] and 41% in another study using ultrasound examination [9], although only a minority (8% in the CT study) were symptomatic. When symptomatic, hepatic vascular malformations associated with HHT typically present in later adulthood as high output heart failure, due to significantly increased blood flow through shunting pathways in the liver [10]. Intracranial hemorrhage is the risk posed by brain AVMs, which are typically congenital and occur in about 10% of those with HHT [11,12].

The clinical diagnosis of HHT is based on the Curaçao Criteria: (i) recurrent and spontaneous nosebleeds (epistaxis), (ii) cutaneous or mucosal telangiectases on the skin of the hands, lips, or face, or inside of the nose or oral cavity, (iii) visceral AVMs or telangiectases in one or more of the internal organs, including lungs, brain, liver, gastrointestinal tract, and spinal cord, and (iv) family history of HHT (i.e., first-degree relative with a definite HHT clinical or genetic diagnosis). The HHT diagnosis is considered definite if three criteria are present, possible or suspected with two criteria, and unlikely if only one is present [3,13]. It is of note that the disease experts who initially created and twenty years later confirmed these consensus clinical criteria for HHT, believe that the locations of telangiectases and AVMs are specific in HHT. In fact, multiple telangiectases or an AVM in a location other than those considered characteristic actually argue against the diagnosis of HHT.

Overall, the phenotype and age of onset of manifestations is highly variable in HHT and this variability seems to depend on HHT subtype, as well as genetic background and/or environmental triggers (second-hits) to which each individual is exposed.

## 2. Genetics of HHT: The Germline Mutation

Heterozygous mutations in several genes are known to cause HHT. Endoglin (*ENG*) mutations cause what is referred to as HHT1 (OMIM #187300) [14], activin receptor-like kinase 1 (*ACVRL1*) mutations cause HHT2 (OMIM #600376) [15], while mothers against decapentaplegic homolog 4 (*MADH4* or *SMAD4*) mutations cause a syndrome which combines familial juvenile polyposis and HHT (JP/HT; OMIM #175050) [16]. Also, mutations in the *GDF2* gene, encoding bone morphogenetic protein 9 (BMP9), were described as the cause of an HHT-like syndrome [17], also named as HHT5 (OMIM #615506). Two further loci have been found by linkage analyses on chromosomes 5 (HHT3) and 7 (HHT4), but their corresponding coding genes have not been identified [18,19].

*ENG* and *ACVRL1* are the predominant genes mutated in HHT, each responsible for almost half of cases. A mutation in one of these two genes is detected in over 95% of individuals who meet Curaçao diagnostic criteria, and a mutation in *SMAD4* is detected in an additional 1–2% [20,21]. Although the phenotypes generated by mutations in *ENG* or *ACVRL1* are similar enough that they cannot be reliably distinguished in the clinical setting, pulmonary and cerebral AVMs are more frequent in HHT1 patients while GI bleeding and liver AVMs are more common in HHT2 [22,23]. Studies suggest that solid organ AVMs in JP/HHT are at least as common as in HHT1 and HHT2, and that pulmonary AVMs may be more frequent [16,21,24,25]. Overall, Curaçao diagnostic criteria for HHT are highly predictive of a pathogenic variant in *ENG* (HHT1) or *ACVRL1* (HHT2) but cannot distinguish between these two genotypes [21]. Furthermore, the genetic heterogeneity does not explain the striking variable expression observed within families.

There are no common disease-causing mutations or mutation hot spots in any of the HHT genes. Both *ENG* and *ACVRL1* mutations that cause HHT are dispersed almost equally throughout the genes except in a couple of exons. Mutations of all types have been reported [20,26]. The frequency of single or several exon deletions/duplications is up to 10%. Mutations causing sequence changes are slightly more common in the *ENG* than *ACVRL1*. Missense mutations account for more than half of mutations detected, however, nonsense, deletions, insertions, and splice site mutations have also been reported. Mutant *ENG* and *ACVRL1* proteins, including the products of multiple missense mutations, were shown to be expressed at the 50% level, in accordance to the haploinsufficiency model [20].

**Figure 1:**
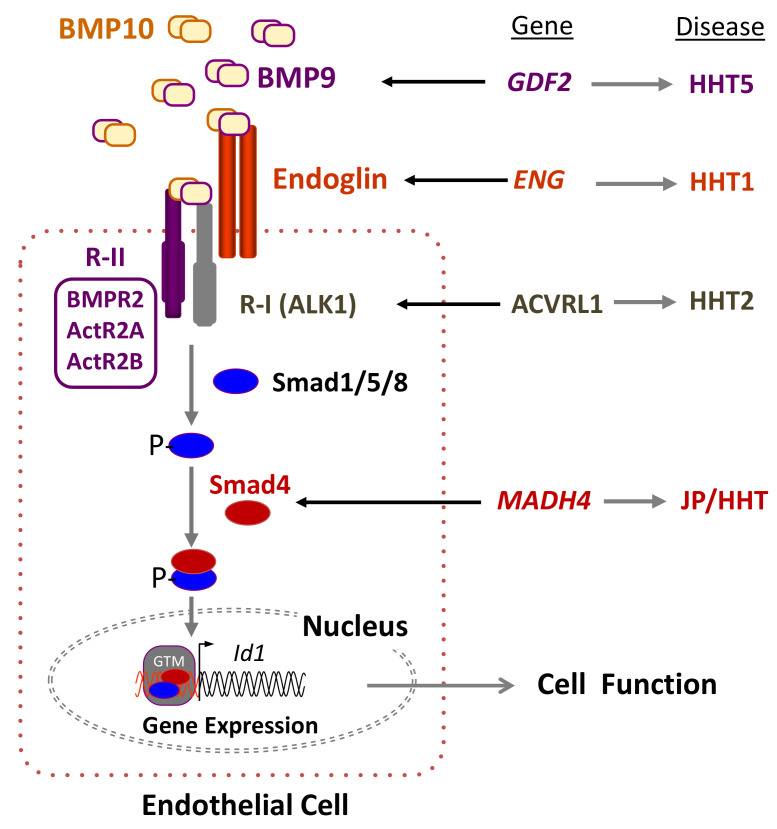
Hereditary hemorrhagic telangiectasia (HHT) and the transforming growth factor beta (TGF-β) signaling pathway in endothelial cells. Heterodimers of bone morphogenetic protein 9 (BMP9) and BMP10, among other members of the TGF-β family, bind to an endothelial cell surface receptor complex composed by the type I (R-I) receptor named ALK1 and the type II (R-II; BMPR2, ActR2A, ActR2B) receptor, both serine/threonine kinases, as well as the auxiliary receptor endoglin. The heterodimeric association between different R-I and R-II determines the specificity of the ligand signaling. Upon ligand binding, the R-II transphosphorylates ALK1, which subsequently propagates the signal by phosphorylating the receptor-regulated Smad (R-Smad) family of proteins, Smad1/5/8. Once phosphorylated (P-), R-Smads form heteromeric complexes with a cooperating homologue named Smad4 and translocate into the nucleus, where they regulate the transcriptional activity of different target genes, in turn modulating endothelial cell function. The involvement of other components of the TGF-β pathway has been omitted for simplification [32]. BMP9, Endoglin, ALK1, and Smad4 proteins are encoded by *GDF2*, *ENG*, *ACVRL1*, and *MADH4* genes, whose pathogenic mutations give rise to HHT5, HHT1, HHT2, and JPHT, respectively. BMP, bone morphogenetic protein; GTM, general transcription machinery. Adapted from Ruiz-Llorente et al. [34].

All the genes mutated in HHT encode proteins involved in the signaling pathway of the transforming growth factor beta (TGF-β) superfamily, including bone morphogenetic proteins (BMPs) (Figure 1). The most likely affected pathway in HHT involves the auxiliary receptor endoglin associated with the signaling serine/threonine kinase receptor ALK1. Both proteins are able to bind the ligands BMP9 and BMP10 [27,28,29], which form a heterodimeric complex that provides most of their BMP biological activity in plasma [30,31]. Upon ligand binding, ALK1 phosphorylates Smad1/5/8 followed by their nuclear translocation in complex with Smad4 [32,33,34,35]. Because endoglin and ALK1 are predominantly expressed in endothelial cells, which respond to circulating BMP9, they are widely accepted as the target cells most affected in HHT.

It is worth noting that within the TGF-β system, endoglin is known to participate in several receptor complexes, not necessarily including ALK1, and is capable of binding different ligands [36,37,38,39]. In addition, endoglin has been reported to be involved in several pathways relevant to vascular homeostasis. Endoglin was shown to regulate the organization of the actin cytoskeleton in endothelial cells via the interaction of its cytoplasmic domain with zyxin and zyxin-related protein 1 (ZRP1) [40,41]. Upon interacting with these members of the zyxin family, endoglin coordinates matrix-dependent cues with actin dynamics like stress fibers and focal adhesions. In primary cultures of endothelial cells from HHT1 patients, endoglin deficiency appears to lead to a disorganized F-actin cytoskeleton and abnormal tube formation [42]. This is in agreement with the fragile mucocutaneous telangiectases that easily break, leading to the frequent nose and gastrointestinal bleedings present in HHT patients. An active search for novel endoglin-specific interactors has allowed the identification of multiple proteins, suggesting the involvement of endoglin in ALK1-independent pathways; nonetheless, the functional characterization and relevance of the novel interactors to the HHT1 field remains to be established [39,43]. Overall, it can be postulated that the phenotypic differences between HHT1 and HHT2 could arise, at least in part, from a subthreshold endoglin participation in these ALK1-independent signaling pathways.

## 3. Pathogenic Mechanisms in HHT: The Second-Hit Hypothesis

A deficient expression of the HHT genes underlies the molecular basis of disease pathogenesis. Mono-allelic loss of expression leading to haploinsufficiency of the respective HHT1 and HHT2 proteins has been shown to dysregulate TGF-β/BMP signaling in endothelial cells negatively impacting cell proliferation, migration, and recruitment during vascular remodeling and angiogenesis [20,34,44,45].

Different *Eng* or *Acvrl1* genetic mouse models of HHT have been described by several groups during the last three decades [45]. Mice lacking functional endoglin [46,47,48] or ALK1 [49,50] were generated by germline gene-targeting. In all cases, global embryonic loss of endoglin or ALK1 expression leads to cardiovascular defects, enlarged fragile vessels, and embryonic lethality by mid-gestation. These early models suggested that both endoglin and ALK1 were essential for cardiovascular development and homeostasis and that a further decrease in the level of these essential proteins could lead to vascular abnormalities.

The second-hit hypothesis, also known as Knudson hypothesis, was first described to explain the progression of cancer [51]. It was suggested that the first event of tumorigenesis in familiar cancers would be due to germline inactivation of one allele, followed by somatic inactivation of the second allele. Recently, using next-generation sequencing, Snellings et al. [52] have been able to demonstrate the presence of low-frequency somatic mutations in telangiectases of HHT1 and HHT2 patients, suggesting that the bi-allelic loss of *ENG* or *ACVRL1* was required for the development of vascular lesions. Overall, haploinsufficiency is widely accepted as the underlying cause of HHT1 and HHT2 pathogenicity [20,34,53], although a dominant negative effect has been described in a few *ENG* and *ACVRL1* pathogenic mutations [54,55,56,57]. Nonetheless, neither haploinsufficiency nor a dominant negative effect by itself can account for the localized generation of vascular lesions in HHT patients. It is intriguing that the vascular HHT lesions appear only at distinct sites within certain organs, rather than being present throughout the body and in all organs/tissues. This paradox has been explained, as in many other genetic diseases, by postulating the need for a second-hit.

**Figure 2:**
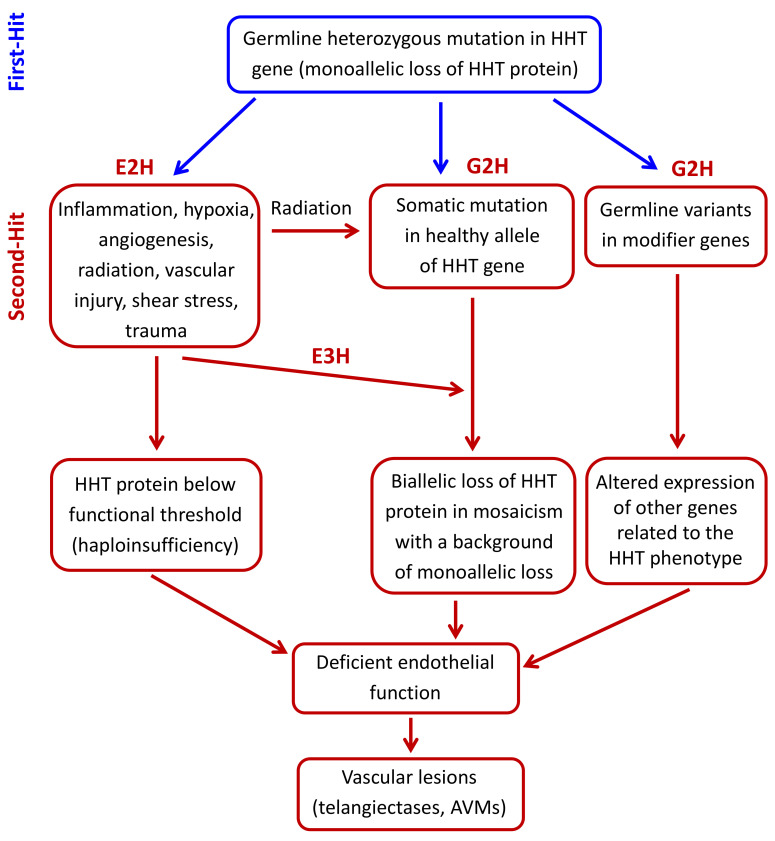
Hypothetical second-hit model in hereditary hemorrhagic telangiectasia (HHT). The germline heterozygous mutation in the HHT gene leads to a monoallelic loss of the encoded HHT protein in endothelial cells (First-hit). A subsequent environmental stimulus like inflammation, hypoxia, neoangiogenesis, vascular injury, radiation, shear stress, or trauma (environmental second-hit; E2H), can induce the expression/activation of mediators, which generate a microenvironment where HHT protein levels are below the needed functional threshold. This drop in the HHT functional protein can also be generated by a somatic mutation in the normal allele (genetic second-hit; G2H), leading to a focal protein loss in lesions. One possible cause of somatic mutation is sunlight radiation, especially in skin telangiectases. A somatic mutation could also synergize with an environmental third-hit (E3H). Modifier genes (G2H) could also contribute to focal vascular lesions by affecting HHT protein level and activity. In all cases, the result is an impaired endothelial cell function, leading to the generation of telangiectases or arteriovenous malformations (AVMs).

The second-hit hypothesis has evolved over the years in order to explain how multiple factors, either environmental or genetic (modifier genes or somatic mutations), can contribute to complex diseases with phenotypic heterogeneity. In the case of HHT, external or physiological triggers such as vascular injury, inflammation, and angiogenic stimuli could account for the generation of AVMs. We will first describe the environmental factors proposed for HHT and their effects on endothelial cell functions. We will then describe how modifier genes or a somatic mutation in the normal HHT allele may synergize with a background of deficient endoglin or ALK1 expression/activity to generate vascular lesions [58] (Figure 2).

### 3.1. Environmental Second-Hits

The underlying pathogenic mechanisms leading to new telangiectases and AVMs in HHT are not completely understood. The variability in severity of symptoms and age of onset amongst patients, even within members of the same family, suggests that the HHT phenotype involves the contribution of environmental factors, which can be external or physiological.

#### 3.1.1. Mechanical and Light-Induced Triggers

It is widely recognized that disease progression worsens with a patient’s age [1,59]. An increased number of telangiectases in hands and lips from older patients have been observed, a finding compatible with the assumption that aged patients have been subjected to more cutaneous insults over time than younger patients. To further confirm this hypothesis, a recent study has assessed the influence of environmental triggers (mechanical or light-induced) on the number of cutaneous telangiectases in HHT patients, taking into account their dominant hand and exposure to sunlight [60]. Overall, HHT patients developed more telangiectases on their dominant hand, suggesting that mechanical stress induced by manual work may account for this increase. They also developed more telangiectases on their lower lip than on the upper lip. This preferential location could be explained by the fact that the lower lip is subjected to more physical damage than the upper lip; for example, it is in contact with the upper incisor teeth, and gravity naturally increases the chances of food touching the lower lip. In addition, HHT patients, who claimed to have had excessive sun exposure in the past, exhibited a higher number of telangiectases on both lips. While sunlight comprises a spectrum of light with varying wavelengths (ultraviolet (UV), visible, and infrared), the induction of telangiectases is likely caused predominantly by the damaging UV light with shorter wavelength (100–400 nm) [60]. Taken together, these results show that mechanical and sunlight-induced trauma strongly influence the formation of telangiectases in HHT patients, suggesting potential implications in preventive measures for HHT [60].

The effect of tissue wounding, mechanical stimulation, or radiation has been analyzed in several HHT animal models. Upon skin wounding, conditional knockout mouse models, in which both copies of *Eng* [61], *Acvrl1* [62], or *Smad4* [63] were postnatally deleted, developed vascular malformations. Also, it has been reported that defective fluid shear stress mechano-transduction mediates the formation of AVMs in HHT2 animal models [64,65]. The underlying mechanism appears to involve the BMP9/endoglin/ALK1/Smad4 pathway [66]. Thus, ALK1 expression requires blood flow [64], and fluid shear stress potentiates BMP9 activation of ALK1 signaling, which correlates with enhanced association of ALK1 and endoglin. It is noteworthy that vascular injury stimulates gene expression of endoglin and ALK1 via activation of the stress-inducible transcription factor KLF6 (Kruppel-like factor 6) [67,68], suggesting the protective functional involvement of endoglin and ALK1 under stress in healthy conditions. In fact, endoglin modulates shear-induced collateral artery growth and prevents vascular malformation by regulating flow-induced cell migration and specification [69,70]. Moreover, ALK1 is needed for BMP9 and flow responses and mediates both inhibition of endothelial proliferation and recruitment of mural cells, favoring vascular stabilization [65]. Thus, it is expected that a decreased endoglin or ALK1 expression in HHT would be detrimental for the vasculature under shear stress conditions.

Furthermore, SMAD4 is an essential effector of BMP9/ALK1 signaling that affects AVM pathogenesis via regulation of casein kinase 2 and its expression prevents flow-induced AVMs [71]. The role of endoglin in response to flow was also analyzed using zebrafish embryonic development as a model to study blood vessel expansion and contraction [72]. Interestingly, in loss of function endoglin mutants, blood vessels abnormally enlarge in response to flow and exacerbate pre-existing embryonic arterial-venous shunts, suggesting that endoglin controls blood vessel diameter in response to hemodynamic cues [73]. In addition, a subset of HHT patients present with polymicrogyria, a condition characterized by abnormal development of the brain with vascular regions experiencing low fluid shear stress during corticogenesis in utero, leading to a brain surface with many ridges or folds, called gyri. In HHT, polymicrogyria appears exclusively associated with a subset of pathogenic variants in endoglin that is involved in blood flow-related mechano-transduction [74]. Taken together, these findings suggest that the interplay between the BMP9/endoglin/ALK1 pathway and blood flow-induced mechano-transduction signals plays a critical role during development of HHT lesions [64,65,66,72,73].

The effect of ionizing radiation in the formation of telangiectases has also been analyzed in an HHT1 mouse model [75]. Kidneys of *Endoglin* heterozygous (*Eng^+/−^*) or wild-type mice were irradiated with 16 Gy and mice were sacrificed after 20 weeks. Intriguingly, *Eng^+/−^* mice displayed reduced telangiectasia formation in the irradiated kidney compared to controls [75]. While the nature of the ionizing radiation used and the tissue irradiated were different in these studies, their results are at variance with the expected increased number of telangiectases induced by sunlight in HHT patients [60].

#### 3.1.2. Modulators of Endothelial Function

The major HHT gene products (endoglin and ALK1) are predominantly expressed on endothelial cells which are recognized as the target cells in this disorder. Therefore, HHT-induced changes in endothelial cell function, including the monoallelic loss of expression of either HHT gene, are likely to impact vascular homeostasis and the response to external environmental hits as well as to physiological stimuli, leading to lesions such as telangiectases and AVMs.

##### VEGF-Dependent Angiogenic Stimuli

The Vascular Endothelial Growth Factor (VEGF) family of growth factors targets endothelial cells by preferentially binding to VEGF receptor 1 (VEGFR1), stimulating cell proliferation and migration and thereby promoting angiogenesis and vascular remodeling. The wound-induced de novo AVM formation in HHT animal models involves angiogenic processes with active extension of arterial blood vessels, meeting growing venous branches [61]. Furthermore, VEGF levels were shown to be elevated in skin telangiectases and plasma of HHT patients [76,77,78], leading several research teams to investigate the role of VEGF in the generation of HHT vascular lesions. The delivery of recombinant human VEGF165 (AdhVEGF) into basal ganglia led to increased micro-vessel density in both *Eng* heterozygous (*Eng^+/−^*) and *Eng^+/+^* mice, as expected from the pro-angiogenic activity of VEGF. However, confocal microscopic examination revealed grossly abnormal micro-vessels in *Eng^+/−^* mouse brains that were not observed in *Eng^+/+^* mice. Abnormal micro-vessels featured enlargement, clustering, twist, or spirals [79]. Similar results were obtained using a Cre transgenic mouse line where *Eng* was deleted in smooth muscle and endothelial cells. Ectopic expression of VEGF into the brain to induce focal angiogenesis promoted the formation of AVMs [80]. These two HHT1 brain AVM models show that VEGF induces AVMs in the *Eng* heterozygous adult mouse brain, suggesting that VEGF stimulation may play a pivotal role in the initiation and development of vascular malformations in states of endoglin insufficiency present in HHT1 patients [79,80].

The triggering role of VEGF in AVM formation was also observed in *Acvrl1*-deficient mice; although, comparative studies showed that upon angiogenic stimulation with VEGF, deletion of *Eng* induces a more severe cerebrovascular dysplasia per copy than that of *Acvrl1* [62,81]. Also, a distinct pulmonary and hepatic angiogenic profile and response to anti-VEGF treatment was found between *Eng* and *Acvrl1* heterozygous mice [82]. It is noteworthy that in wound-induced skin AVMs of *Acvrl1*-deficient adult mice, VEGF neutralizing antibody can prevent AVM formation and ameliorate internal bleeding. In addition, with topical applications at different stages of AVM development, the VEGF blockade can prevent both the formation of AVMs and cease their progression [62]. These observations not only show that VEGF-dependent angiogenesis is a key event during AVM formation, but also suggest that VEGF inhibition could be an effective therapy for prevention of AVM development. In fact, Bevacizumab (Avastin), a humanized monoclonal antibody to VEGF and potent anti-angiogenic drug, has been used to treat HHT patients with severe epistaxis, GI-bleeding, or high-output cardiac failure/hepatic AVMs [83].

##### ALK1 Signaling and the Notch Pathway

In recent years, an increased interest has been devoted to the crosstalk between endothelial ALK1 and Notch in the development of AVMs and their possible involvement in HHT [84,85]. The Notch pathway is an intercellular signaling pathway, where both receptor and ligand are membrane-bound on adjacent cells. Signaling initiates when cell surface Notch receptors engage ligands like Delta-like 4 (Dll4) or Jagged1 (JAG1) on opposing cells, leading to the cleavage of Notch and the release of Notch Intercellular Domain (NICD) into the cytoplasm. Then, NICD translocates into the nucleus to initiate the transcription of the downstream targets HEY1 and HEY2, transcriptional repressors involved in VEGF-dependent signaling and arterial cell fate, among others [85,86]. Previous studies had shown that arteriovenous shunts occur in mouse and zebrafish mutants for components of the Notch signaling pathway [87,88], a finding that prompted the investigation of a role for the Notch pathway in AVM pathogenesis of HHT [84]. In *Acvrl1* knockout mouse models of HHT2, AVMs show decreased Notch signaling with loss of ALK1 causing expansion of the shunt through endothelial cell proliferation. Also, by cooperating with the Notch pathway, expression of ALK1 inhibits angiogenesis, and blocking ALK1 signaling during postnatal development in mice leads to retinal hypervascularization and the appearance of AVMs. Furthermore, combined blockade of ALK1 and Notch signaling exacerbates hypervascularization, whereas activation of ALK1 by its high-affinity ligand BMP9 rescues hyper-sprouting induced by Notch inhibition. The molecular basis of this regulation appears to involve ALK1-dependent Smad signaling in synergy with activated Notch in stalk endothelial cells to induce expression of the Notch targets HEY1 and HEY2, which, in turn, repress VEGF signaling, tip endothelial cell formation, and endothelial sprouting [84]. These results demonstrate a direct link between ALK1 and Notch signaling routes during vascular morphogenesis that may be relevant to the pathogenesis of HHT [85]. It can be postulated that dysregulation of ALK1 signaling, achieved upon ALK1 or endoglin haploinsufficiency, may act as a second-hit, impairing the ALK1/Notch collaboration and leading to the formation of the vascular lesions in HHT.

##### Proliferation and Apoptosis Stimuli

Endoglin is markedly upregulated in the proliferating endothelium of tissues undergoing angiogenesis. This is in agreement with the fact that the endoglin/ALK1 pathway promotes endothelial cell proliferation and opposes TGF-β1/ALK5-dependent responses, including inhibition of cellular proliferation [58,89]. Furthermore, neutralizing anti-endoglin antibodies or silencing endoglin enhances the inhibitory effect of TGF-β on proliferation and migration, whereas ectopic endoglin expression counteracts the anti-proliferative effect of TGF-β1 in endothelial cells [90,91]. Moreover, inhibition of endoglin expression on endothelial cells increases the anti-proliferative effect of TGF-β1 and enhances endothelial cell apoptosis induced by hypoxia and TGF-β1 [90,92]. It is worth noting that apoptosis can be induced by chemotherapeutic drugs or UV irradiation [93], with UV light being one of the potential second-hits described in HHT patients [60]. These results demonstrate that the endoglin/ALK1 pathway not only promotes proliferation, but also counteracts the hypoxia/TGF-β-induced apoptosis of endothelial cells. This role in endothelial cell survival suggests that in the presence of anti-proliferative and/or pro-apoptotic stimuli, decreased activity of the endoglin/ALK1/Smad4 route, as occurs in HHT patients, may lead to reduced cell proliferation and/or apoptosis in capillaries, leading to the vascular lesion. Conversely, the endothelial proliferation associated with triggers of angiogenesis, such as vascular injury, ischemia, or trauma, may synergize with the loss of expression of HHT genes as their products may not reach the necessary threshold to cope with the vascular remodeling required [58]. It should be noted that BMP9 and ALK1 have been described both as pro- and anti-angiogenic factors depending on the cellular context [84,89,94,95]. In this regard, BMP signaling strongly induces expression of the helix-loop-helix transcription factor Id1, which promotes endothelial proliferation and migration [96]; but, paradoxically, BMP9 has been reported to inhibit VEGF- and fibroblast growth factor (FGF)-induced proliferation [27,97] and has been described as a vascular quiescence factor.

##### Inflammation and Endothelial Cell Adhesion and Nitric Oxide Regulation

Inflammation is a complex biological response to harmful stimuli, such as pathogens, tissue damage, or irritants, involving blood vessels, immune cells, and molecular mediators. The inflammatory context is associated with an upregulated expression of endothelial endoglin, and with an inflammatory cell infiltrate [58,98]. It is noteworthy that HHT skin telangiectases and internal AVMs show a perivascular mononuclear cell infiltrate, including lymphocytes and monocytes/macrophages [99,100], suggesting that both endoglin function and leukocyte infiltration are involved in the vascular repair/remodeling process whose dysregulation may lead to AVM formation in HHT. Indeed, it has been reported that endoglin plays a crucial role in leukocyte-mediated vascular repair in HHT [101], in agreement with the pro-active role of leukocyte infiltration during angiogenesis and vascular remodeling [102]. Several in vivo and in vitro models of inflammation and vascular repair have shown that *Endoglin* heterozygosity leads to an abnormal leukocyte infiltration and function [82,101,103,104,105,106,107]. In a mouse model of dextran sodium sulfate (DSS)-induced chronic colitis, increased leukocyte infiltration of the gut and more severe colitis was observed in *Eng^+/-^* relative to control mice [82,103,105]. Also, upon myocardial infarction, a greater deterioration in cardiac function was observed in *Eng^+/−^* compared to control mice, although host inflammatory leukocyte numbers in the infarct area were similar; however, defects in vessel formation and heart function in *Eng^+/−^* mice were rescued by injection of leukocytes from healthy human donors, but not by leukocytes from HHT1 patients [101]. Using a distal middle cerebral artery occlusion model, *Eng^+/−^* mice showed larger infarct/atrophic volumes associated with fewer infiltrating macrophages, suggesting that endoglin deficiency impairs brain injury recovery by inhibiting macrophage homing, delaying inflammation resolution, and reducing angiogenesis [106]. In addition, decreased inflammation-induced leukocyte trafficking to the peritoneum and lungs was found in *Eng^+/−^* mice treated with the inflammatory stimuli carrageenan or lipopolysaccharide (LPS), respectively [104].

The underlying molecular mechanism by which endothelial endoglin is involved can be explained at least in part by its capacity to act as a counter-receptor of leukocyte integrins [104,108,109], thus regulating not only endothelium-leukocyte adhesion, but also leukocyte extravasation [104,110]. These processes appear to be mediated by pro-inflammatory molecules such as the chemokine CXCL12, an integrin activator, which strongly promotes leukocyte adhesion to endothelial endoglin or to purified endoglin. Moreover, both endoglin-dependent cellular adhesion and transmigration processes involve the leukocyte integrin α_5_β_1_ via the endoglin arginine-glycine-aspartic acid (RGD) motif [104]. Based on these results, it can be postulated that the function of endothelial endoglin as an adhesion counter-receptor for leukocyte integrins is involved in HHT1 pathogenesis. According to this hypothetical model, in healthy subjects, the capillary network subjected to an inflammatory stimulus is infiltrated with leukocytes that contribute to the vascular repair/remodeling. By contrast, in HHT1 patients, deficient endoglin expression impairs leukocyte infiltration, leading to defective vascular repair/remodeling. As a consequence, the capillary network would disappear and only a preferential channel would remain to eventually become the arterio-venous shunt [110]. While these results may explain the role of endothelial endoglin in leukocyte adhesion within the HHT1 context, the putative cell adhesion-related function of the other HHT gene products, especially ALK1, remains to be explored. Because the kinase ALK1 can interact and target the endoglin cytoplasmic domain for serine and threonine phosphorylation, it can be speculated that this phosphorylation may activate or enhance the binding of endoglin to integrins by inside-out signaling [111,112]. Interestingly, the expression of integrin β_8_ subunit, whose complex with the integrin alpha V binds ligands via the RGD motif, is reduced in sporadic human brain AVMs. In addition, focal deletion of integrin β_8_, combined with an angiogenic VEGF stimulus, enhances vascular dysplasia and hemorrhage in the brain of adult *Acvrl1* heterozygous mice [113]. Further studies are needed to assess the involvement of ALK1 in integrin-dependent cell adhesion.

Inflammation and oxidative stress induced by reactive oxygen species (ROS) are closely related pathophysiological processes in cardiovascular disease [114]. Tissue injuries and infections activate the immune response by infiltrating circulating mononuclear cells into tissues where they can release ROS, in turn stimulating inflammation. ROS are reactive derivatives of O_2_ metabolism that reduce levels of the vasodilator nitric oxide (NO). In endothelial cells, the major enzymatic sources of ROS are respiratory enzymes of the mitochondria, nicotinamide adenine dinucleotide phosphate (NADPH) oxidases, and uncoupled endothelial nitric oxide synthase (eNOS). Several lines of evidence support the involvement of endoglin and AlK1 in the regulation of eNOS-derived ROS. Endoglin positively regulates the expression and function of eNOS [39,115,116] and forms a complex with eNOS in caveolae, providing a stabilizing function for eNOS. Upon Ca^2+^-induced activation, *Eng^+/−^* endothelial cells show reduced eNOS/Hsp90 association, produce less NO, and generate more eNOS-derived superoxide (O^2−^), indicating that endoglin is an important regulator in the coupling of eNOS activity. Resistance arteries from *Eng^+/−^* mice display an eNOS-dependent enhancement in endothelium-dependent dilatation and impairment in the myogenic response, and treatment with an O_2_^−^ scavenger reverses these vasomotor abnormalities [116]. Both endoglin and ALK1 haploinsufficiency lead to eNOS-derived ROS, oxidative stress, and endothelial dysfunction, with potential pathogenic consequences in HHT [117,118]. Relatedly, to reduce telangiectasia-derived bleeding in HHT patients, therapeutic interventions with the antioxidant, N-acetyl cysteine, aiming to decrease ROS bioavailability, have yielded promising results [119,120].

### 3.2. Genetic Second-Hits

#### 3.2.1. Germline Modifier Variants/Genes

Modifier genes are those in which a genetic variation modifies the effects of mutation at a major gene locus and in so doing, affect disease severity. That modifier genes affected the phenotype in HHT was initially suggested by the study of heterozygous *Eng* and *Acvrl1* mice. The first animal model of HHT was generated in *Eng* heterozygous (*Eng^+/−^*) mice of the 129/Ola inbred strain that were subsequently backcrossed onto the C57BL/6 strain [46]. Early observations of these *Eng* heterozygous mice revealed that disease manifestations were associated with the 129/Ola background, suggesting that other gene(s) contributed to the generation of vascular lesions. Analysis of a large number of mice over a period of one-year established disease prevalence at 72% in 129/Ola, intermediate in backcrosses (36%), and low in C57BL/6 (7%) [121]. Multiple signs of HHT were detected, such as ear telangiectasia, hemorrhage, dilated vessels, liver and lung congestion, brain and heart ischemia, and even cerebral AVMs [121,122]. Disease sequelae included stroke, fatal hemorrhage, and congestive heart failure. Interestingly, 129/Ola inbred mice had previously been shown to carry significant alterations in liver and lung vasculature, such as portal shunting and reduction/truncation of peripheral vessels, when compared to C57BL/6 mice [123,124]. These vascular features might have contributed to the more severe HHT manifestations observed in the *Eng^+/−^* 129/Ola mice. These results strongly suggested that the genetic background via alterations of pathways critical to vascular function in the context of reduced endoglin (and/or ALK1) expression can modify the outcome of disease [44].

Similar to the HHT1 animal model, *Acvrl1* heterozygous mice have dilated vessels and HHT-like vascular lesions in liver, nailbed, intestine, or skin that develop between 7 and 20 months [125]. However, these lesions occur with partial penetrance (approximately 40%). Both *Eng^+/−^* and *Acvrl1^+/−^* adult mice present normal blood vessels, suggesting there are no major vascular defects during developmental angiogenesis [45]. This finding agrees with the observation that the majority of the vasculature in HHT patients functions normally while vascular lesions are localized and sporadic. In addition, animal models with systemic or endothelial-specific deletion of *Eng*, *Acvrl1*, or *Smad4* in adulthood lead to vascular malformations compatible with an HHT phenotype [45,61,63,80,126,127,128].

The search for human modifier genes of HHT was initiated by looking for genetic regions syntenic to the mouse modifier loci, *Tgfbm’s*, that modify the lethal vascular phenotype of *Tgfb1^−/−^* mice [129]. The rationale was that endoglin is a co-receptor for TGF-β1 and that *Eng* null mice have an embryonic lethal phenotype similar to that of the *Tgfb1* null mice. This study led to the identification of human polymorphic variants of the protein, non-receptor tyrosine phosphatase 14 (PTPN14), in the *Tgfbm2* region, showing genetic association with the presence of PAVMs in HHT1 and HHT2 patients in two independent populations. PTPN14 was shown to modulate angiogenesis in endothelial cell culture and to alter the expression of EphrinB2, important in arteriovenous specification [129].

It was subsequently shown that genetic variation within the functional *ENG* allele inherited from the non-affected parent was associated with the presence of pulmonary AVMs in HHT1 patients [130]. Expression of the pulmonary AVM at-risk *ENG* variant, *rs10987746*-C, correlated with *ENG* mRNA levels in a panel of lymphoblastoid cell lines. Furthermore, expression quantitative trait loci (eQTL) analysis showed association between the *rs10987746*-C variant and higher expression of *PTPN14* in a panel of angiogenically active lung adenocarcinoma samples, but not in normal lungs. Quantitative TaqMan^®^ expression analysis in a panel of normal lung tissues from genetically heterogeneous interspecific backcross mice, demonstrated a strong correlation between expression levels of *Eng*, *Acvrl1*, and *Ptpn14*, further suggesting a related role for these genes in lung biology [130]. *PTPN14* has also been shown to be a negative regulator of Yap/Taz signaling, implicated in mechano-transduction, suggesting a potential link between endoglin/ALK1 signaling and shear stress [130].

Variants of ADAM17, which maps within the *Tgfbm3* region, have also been shown to associate with the presence of PAVMs in HHT1 but not HHT2 [131]. ADAM17 is known to downregulate Smad2 signaling by shedding the extracellular domain of TGF-β receptor I (ALK5), and therefore would not affect an ALK1-dependent pathway.

Common polymorphisms in HHT genes other than the disease-causing mutation can be associated with differences in HHT phenotype severity, and specifically, the presence of AVMs. For example, the *ACVRL1* *c.314-35A>G* common variant was shown to be associated with sporadic brain AVMs [132,133], and also subsequently with PAVMs as well as vascular malformations overall in patients with *ENG* mutations, but not in patients with *ACVRL1* mutations [134].

Furthermore, a recent analysis found the number of deleterious variants in angiogenesis-related genes to be significantly higher in HHT patients versus healthy individuals. A comparison of the frequencies of variants in these angiogenesis-related genes in a 100-exome dataset from HHT samples with those from publicly available population frequency datasets suggests that the combination of several modifying variants contribute to the phenotypic heterogeneity of HHT (Bayrak-Toydemir et al., unpublished data). Another study analyzed 11 candidate variants of *Tgfbm* loci in 752 HHT patients and could not find any association with the presence of PAVMs. They also did not find significant associations between variants reported in sporadic AVMs and vascular malformations in HHT [135]. Further investigations are needed to elucidate the role of modifier genes in the phenotypic heterogeneity of HHT. It is likely that such a complex disorder is influenced by genetic modifiers, as has been observed in other diseases such as cystic fibrosis [136,137].

#### 3.2.2. Somatic Mutations in the Second Allele of the HHT Genes

The original second-hit hypothesis refers to a familial form of cancer caused by the germline inactivation of one allele, followed by the somatic inactivation of the second allele [51]. In the context of a germline mutation, random somatic mutations are much more likely to lead to biallelic loss, explaining the multiple tumors that characterize familial cancers. The second-hit mechanism is accepted for tumors and has also been reported for vascular malformations. The first vascular malformation syndrome in which a somatic second-hit was described is the cerebral cavernous malformation (CCM) [138]. Familial CCM samples carry a somatic second mutation in one of the *CCM* genes [139]. Mouse models also support this mechanism, as the loss of both alleles of a *CCM* gene is required for CCM lesions [140]. In the last several years, there have been multiple publications demonstrating the somatic second-hit as part of the disease mechanism to explain the development of multifocal vascular lesions. These include somatic mutations in *RASA1* in lesions from patients with capillary malformation-arteriovenous malformation (CM-AVM) and *TIE2* (*TEK*) somatic mutations in lesions from patients with venous malformations as predominant examples [141,142,143].

The ability to detect somatic mutations in very low frequency has been aided by the advent of next-generation sequencing (NGS). Using NGS, Snellings et al. [52] have been able to demonstrate the presence of low-frequency somatic mutations in telangiectases of HHT1 and HHT2 patients, suggesting that the bi-allelic loss of *ENG* or *ACVRL1* was required for the development of vascular lesions. Somatic mutations in either *ENG* or *ACVRL1* could be identified in skin telangiectasia tissue from a small number of patients with HHT [52]. They studied 3 mm skin biopsies from telangiectases obtained from one patient with an *ENG* germline mutation and 4 patients with different *ACVRL1* mutations. Somatic mutations were only identified in 47% of samples (9 of 19 samplings from 5 patients) from 4 of 5 patients studied. These second mutations were different from the patient’s germline mutation and were located on the other allele. Dermal telangiectasia tissues showed loss of function mutations with complete loss of the protein function. These second mutations were also different on distinct telangiectasia biopsies of the same individual. Somatic second-hit mutation frequency in the tissue was up to 2.3% on all of these samples. Possible explanations for this low frequency include telangiectases being highly mosaic for the somatic mutation, a high percentage of normal tissue present in the biopsy, or variable cellular composition in distinct biopsies where the genetic second-hit is restricted to a cell type of low abundance within the lesions. The observation that somatic mutations were not seen in every biopsy sample tested may suggest that not all telangiectases carry a somatic mutation and that an environmental tertiary-hit might still be required for a vascular lesion to occur. Of note, the telangiectases used for this study were of the skin, a tissue which undergoes a lifetime of exposure to sunlight radiation, a known mutagen that could generate some of the HHT somatic mutations (Figure 2). Overall, these preliminary observations need to be extended and confirmed in additional genes such as *SMAD4*, and in additional tissues, including visceral AVMs.

The pathogenic mechanism by which the biallelic loss, triggered by a somatic mutation, leads to the HHT phenotype has been experimentally addressed in some knockout animal models. By itself, the loss of function of both alleles does not appear to be sufficient to develop the vascular lesions. Interestingly, in adult knockout mouse models for the three major HHT genes (*Eng*, *Acvrl1*, and *Smad4*), the HHT phenotype appears to develop upon local injury or an angiogenic stimulus, such as VEGF [61,62,63,144]. In addition, the appearance of vascular lesions in embryos and adult *Eng* and *Acvrl1* knockout mice in the absence of external stimuli suggests that internal physiological cues such as angiogenesis or shear stress may also contribute to their HHT phenotype [126,127]. Accordingly, it can be postulated that compared to a monoallelic loss (heterozygous background), the localized biallelic loss generated by a somatic mutation may give rise to a faster and more severe HHT phenotype in the presence of an environmental or physiological additional hit.

## 4. Conclusions

The two-hit hypothesis has been widely accepted for many years as a plausible explanation for the phenotypic variability found in HHT. Here, we have reviewed some of the reported environmental and genetic second-hits (Figure 2). Thus, germline heterozygous mutations in HHT genes (first-hit) result in a monoallelic protein loss in endothelial cells. A subsequent environmental stimulus (second-hit) like inflammation, hypoxia, neoangiogenesis, vascular injury, shear stress, radiation, or trauma, can induce the expression/activation of mediators, which generate a microenvironment where HHT protein levels are below the needed functional threshold. Furthermore, the existence of a genetic second-hit (somatic mutation in the normal HHT allele), combined with an environmental trigger (tertiary-hit), and/or the presence of modifier variants, can set a much lower threshold for disease development. In all cases, the consequence is an impaired endothelial cell function that leads to the generation of telangiectases and AVMs. Overall, the current clinical data, as well as in vivo and in vitro experimental results, support the second-hit hypothesis to explain why certain individuals with HHT genotypes develop earlier and/or more severe clinical phenotypes than other family members. Also, the organ-specific location of telangiectases and AVMs in HHT, and the differing age of presentation for lesions at each site, suggest that the required second-hits may be tissue-specific. However, major gaps in our knowledge remain, particularly in delineating the exact role of the different second-hits that lead to the development of clinically relevant symptoms in HHT, and how this information can be applied in the clinical practice. Further studies to better understand the pathological mechanisms of HHT, including identification of novel potential second-hits, are needed, as well as the translation of this knowledge into preventive clinical measures and treatments.
